# The Challenges and Strategies of Food Security under Global Change

**DOI:** 10.3390/foods13132083

**Published:** 2024-07-01

**Authors:** Raquel P. F. Guiné

**Affiliations:** CERNAS-IPV Research Centre, Polytechnic University of Viseu, 3504-510 Viseu, Portugal; raquelguine@esav.ipv.pt

## 1. The Challenges of Food Security

Food insecurity corresponds to a deficit in households’ access to appropriate food, either in quantity and/or quality, due to limited financial resources or other factors. A related concept of nutrition insecurity is also gaining relevance, and this refers to a lack of access to food that has the adequate nutritional value necessary for a good health status and well-being [1].

Currently, the world faces troubling challenges that greatly impact food/nutritional security. One of the most worrying trends is an increase in the world’s population and the rising number of countries that are food importers owing to their inability to produce the food they need to feed their population. The United Nations World Population Prospects [2] estimates a rise in the world’s population from about 8 billion in 2025 to 10.5 billion by 2100. An increase in the world’s population brings the added challenge of feeding such a large number of people with the Earth’s limited resources. This is only possible with more efficient food production, more sustainable use of natural resources, and the minimization and reutilization of food and agricultural waste [3].

Complying with efficient food production while minimizing environmental impacts is a major issue since the more environmentally friendly agricultural practices, such as organic farming and integrated protection, do not allow the production of food at the same rates as conventional intensive farming practices [4,5]. Nevertheless, organic farming has a decisive role in achieving sustainability in agriculture, meeting a number of the Sustainable Development Goals of the United Nations [6]. In what concerns the minimization of agricultural and food waste and its reutilization, these goals have been at the top of priorities of academics, farmers, industrials, and even consumers in attempts to minimize waste along the whole food supply chain and to find alternative ways to value foods that are discarded [7,8,9,10].

One other challenge is related to the world’s food supply being strongly under pressure due to the combined effects of environmental degradation and climate change. Environmental degradation, such as sterile agricultural land, depleted water tables, and falling grain yields, threatens food security for hundreds of millions of people, especially in more vulnerable regions of the globe [3,11,12]. Climate change has direct impacts on agriculture. Climate-induced drought reduces crop production and lowers yields, while warmer temperatures lead to a higher incidence of plant, livestock, and fish diseases [13,14]. Climate change is dislocating production in some regions, and this trend is expected to increase as the century progresses. The deforestation of tropical rainforests to obtain arable soil is a pivotal issue that contributes to environmental degradation while trying to respond to a need to increase the production of higher amounts of food [15,16,17].

One other challenge is related to a trend of rising populism allied to nationalism, which has increased trade protectionism. This brings huge constraints to the commercialization of foods on a global scale [18,19]. Allied with this challenge, there is the trend for an increase in the number of weak and at-risk states which are poorly equipped to address their food insecurity and, therefore, face social and/or political instability. 

## 2. Wars and Conflicts as Enhancers of Food Insecurity Risks

War zones and political or social conflicts are major drivers for food insecurity, not only regionally but also on a global scale. Taking the example of the Russia–Ukraine conflict, it was evident that it would have a major impact on agricultural production and trade [20]. While Ukrainian wheat, soybean, and maize production fell abruptly in 2022–2023, the global supply chain and food trade were significantly affected, causing scarcity and a consequent increase in the world’s food prices of these commodities [20]. The wheat crisis could thus be alleviated by increasing production and eliminating trade restrictions [21].

At more localized scales, the consequences of wars and conflicts are devastating for the affected populations, like, for example, those derived from the Israel–Palestine conflict in 2024 [22]. Although the food security of Palestinian households in the Gaza Strip was not directly impacted by the 2014 Gaza conflict due to some resilience and adaptive capacity [23], it is also a fact that these diminish with time and with increasing intensity of the conflict, as has happened in 2024 [22].

## 3. Prospects and Future Challenges

Scientists and politicians have to come together to examine global food security challenges and find the correct strategies to mitigate the risks. Resilient systems must be built to better face the adverse consequences of today’s still inappropriate food systems. It is a fact that much has been done so far to achieve better efficacy in food production, utilization of natural resources and minimization of food and agricultural waste, but much more needs still to be achieved for a balanced utilization of resources while producing food able to satiate the increasing world population.

Success can only be achieved through research, implementation, and collaboration in a global strategy to fight a common problem. Individualism, nationalism, and politics tend to encourage too much self-focus on one’s own success and are enemies of global perspectives and collaborative approaches.

Food production, food trade, climate change, environmental degradation, food supply chains and distribution networks, waste management, governance, and food policies are all issues that will have a decisive role in the future of food security/insecurity.
